# Optimization model for multi-products multi-periods multi-suppliers raw-material selection and composition, and order quantity problem with one-year minimum order quantity contract

**DOI:** 10.1016/j.mex.2023.102350

**Published:** 2023-09-22

**Authors:** Mohammad Rizka Fadhli, Saladin Uttunggadewa, Rieske Hadianti, Sri Redjeki Pudjaprasetya

**Affiliations:** a Magister of Computational Sciences Program, Institut Teknologi Bandung, Center for Advance Sciences 4th floor, Jalan Ganesha no. 10, Bandung 40132, Indonesia; b Faculty of Mathematics and Natural Sciences, Center for Advance Sciences 4th floor, Jalan Ganesha no. 10, Bandung 40132, Indonesia; c Center for Mathematical Modeling and Simulation, Gedung Labtek III 1st floor, Jalan Ganesha no. 10, Bandung 40132, Indonesia

**Keywords:** Inventory control, Multi-period multi-product multi criteria raw-material selection, Mix-integer linear programming, Multi-products, multi-periods, multi-suppliers raw-material selection and composition, and order quantity problem

## Abstract

This paper concerns the optimization model for a multi-product multi-period raw-material selection and composition, and order quantity problem faced by a beverage company.

• There are some criteria in raw material selection, which we accommodate all the criteria in the objective function. There are several suppliers, and one of the decision criteria is a one-year minimum order quantity contract between the company and the suppliers. The actual one-year demand for raw materials may deviate significantly from the one-year minimum order quantities.
• We derive a function that can be regarded as a penalty function to maintain the total order quantities in one year to fulfill the minimum one-year order quantity contracts. This penalty function is a part of the objective function and can be relaxed once the one-year minimum order quantity contracts are fulfilled.
• We performed several numerical experiments to check the optimal solutions for various demands and for various objective functions. These experiments show our MILP (Mixed Integer Linear Programming) gives the desired optimal solutions and show the influence of decision criteria on the optimal solution.

## Graphical abstract

**Figure:**
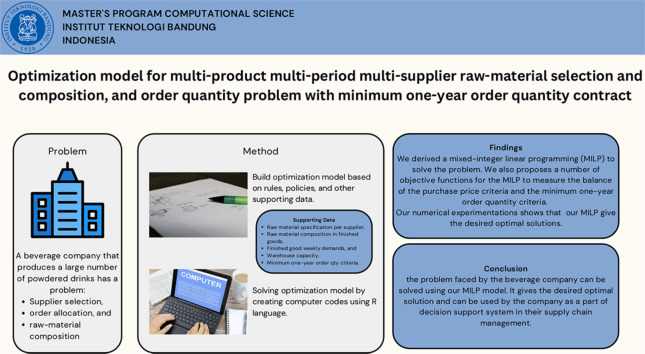

Specifications table

**Table:** 

Subject area:	Mathematics and Statistics
More specific subject area:	Operation Research and Optimization
Name of your method:	Multi-products, multi-periods, multi-suppliers raw-material selection and composition, and order quantity problem
Name and reference of original method:	Multi-products, multi-suppliers, multi periods raw-material selection
Resource availability:	Data can be found in: https://github.com/ikanx101/MILP_Methodx

**Method details**

## Problem

This paper concerns the optimization model for supplier selection, order allocation, and raw-material composition in a beverage company that produces many drink powders. The optimization model is developed in conjunction with the development of a decision support system for monthly decision-making by the company, which involves supplier selection, order allocation, and raw material composition. Prior to implementing this system, the monthly decisions were made manually, leading to significant energy consumption, mainly because the underlying decision-making problem is complex.

There are several suppliers that can provide the same key raw material of the drink powders, but the color or some physical characteristics are slightly different, so we may assume those raw materials are different. The drink powders produced by this company, which in the remainder of this paper are called items, can be classified into two classes of items.

• The first class consists of items that can be produced by using exactly a single type of raw material.
• The second class consists of more flexible items, where each item in this class can be produced using one raw material or a composition of several raw materials. For each item in this class, we then have a set of possible raw materials. The sets of materials may vary from one to the other.

To avoid supply disruption, the company has decided to use multiple sources for these raw materials. The company has established selection criteria for each raw material, which are based on the estimated one-year total demand of raw materials and a subjective assessment of whether the raw material cannot be substituted, price, service, and the minimum order required for each purchase. After determining the score for each raw material, the company decided to make contracts or agreements with six suppliers. Each contract stated the unit price and the minimum order quantity within a year. Based on these contracts, production planning and inventory control of raw materials are carried out.

The estimated total one-year demand for items is obtained from the forecasting process performed yearly. This forecasting process yields the monthly total demand for items, which is timevarying. But at the production level, the company refines the monthly total demand as a response to disruptions such as sudden additional requests due to flash sales practices in e-commerce, and others.

Once the demand for items for a month is issued, the company must make the decision to purchase the raw materials from some suppliers. This purchase decision from a supplier includes the purchase of four serial deliveries one week apart. The first delivery must be no later than 17 days before the following month's start. The 17 days here is the total time required for the company's internal inspection and preparation of raw materials.

This decision process is complex since many items must be produced which mostly belong to the second class, and the monthly demand may vary. Additionally, the company imposes a production regulation for the second class of items because of the multiple-sources policy, which states that each item in the second class must be produced using a composition of at least two types of the corresponding possible raw materials. The decision process must be performed carefully to obtain results in the form of:

• which raw materials are purchased along with the delivery size for every four corresponding weeks,
• the composition of raw materials for every item in the second class which must be produced,

while minimizing the total inventory cost.

The optimization model we derive in this paper can be categorized as a multi-product multi-period raw material selection and composition, and order quantity problem, which we write as a mixed integer linear programming. The optimization model and its solution method are the backbones of the decision support system since it defines the problem space and guides the search for the optimal solution.

## Production planning and inventory control for raw materials

**Fig. 1:**
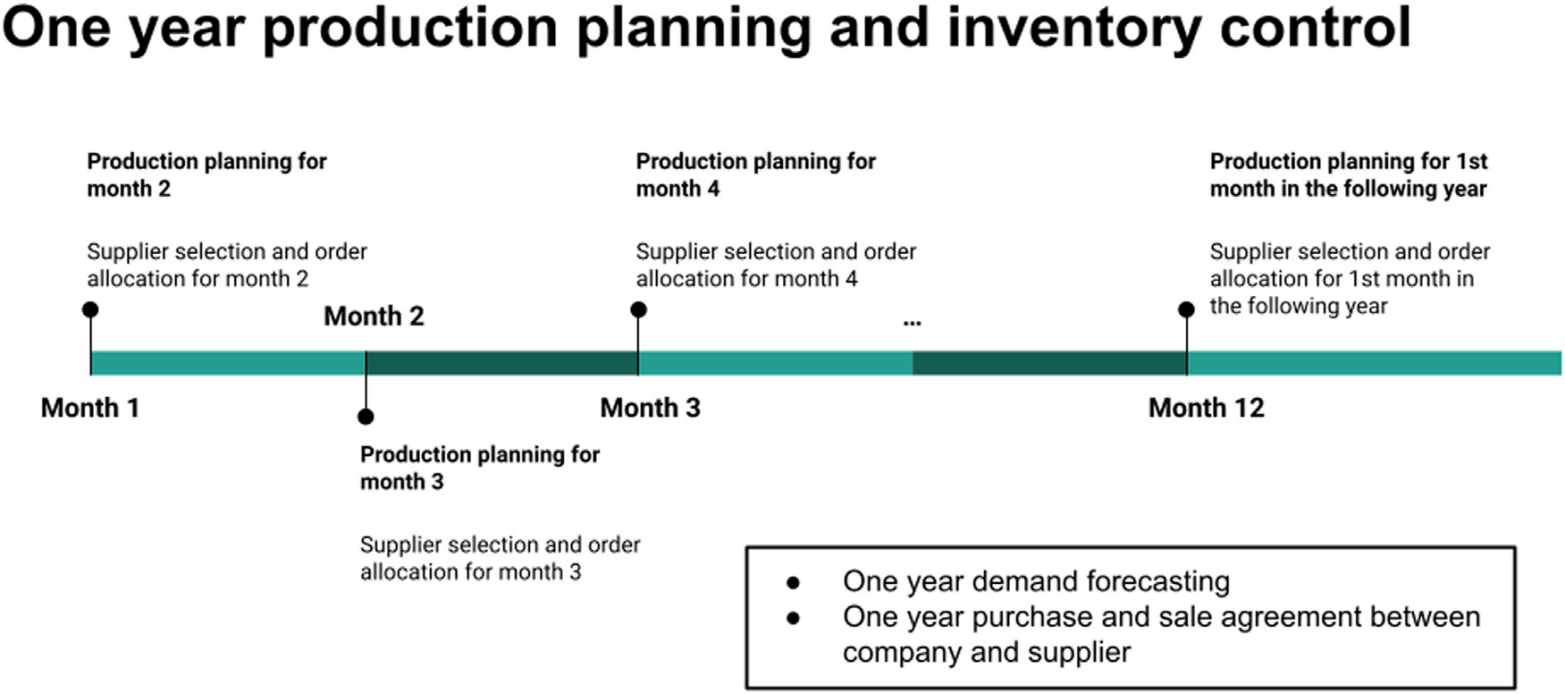
An illustration of the production planning, supplier selection, and order allocation processes in one year.

As mentioned above, the company deals with six raw material suppliers. The decision process for supplier selection and order allocation is carried out every month based on the results of demand forecasting at the beginning of the year and production performance in the previous month. From this demand forecasting process, the company then makes the purchase and sales agreements with all six suppliers concerning the one-year minimum purchase, unit price, and minimum one-month delivery. The serial process in one calendar year can be seen in Fig. 1. At the beginning of the month, the company forecasts the demand for items in the following month.

From the estimated monthly demand for items obtained from the yearly forecasting process, we can directly find out the estimated monthly total demand for raw materials in one year. In practice, this one-month estimated demand must be reviewed due to several things, such as production in the previous month experiencing disruptions, sudden additional requests due to flash sales practices in e-commerce, and others. Reviewing the one-month demand and determining the production schedule we call production planning for one month.

**Fig. 2:**
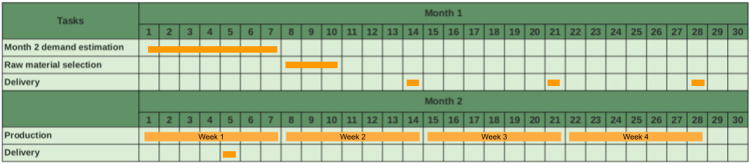
Decision process planning horizon.

As soon as the production planning is performed, the company performs the decision process for purchasing the raw materials from some suppliers. In the following, we assume that one month can be divided into four weeks (the fourth week may be longer than seven days). This purchase decision covers purchases for four serial deliveries one week apart. The first delivery must be no later than 17 days before the following month's start since the internal inspection and the preparation for the raw materials delivered takes 17 days. Fig. 2 in the following illustrates a one-month planning horizon and raw material selection, and four consecutive delivery points follow it.

The decision process considers some parameters for decision making such as:

• raw materials purchase prices,
• existing stock of raw materials in the warehouse,
• the one-month minimum delivery of raw materials (if ordered),
• the one-year minimum purchase of each raw material,
• raw-material flexibility of each item, which is known by the number of raw materials that can be used to produce the item. The larger this number for an item, the more flexible the item,
• and others.

The purchase must also comply with the company's internal policies in the following.

Policy 1 purchase raw materials from at least two suppliers to maintain supply security,

Policy 2 if an item must be produced by using more than one raw material, the proportions of raw materials used are the same.

In the following section, we will accommodate all these policies into some constraints of an optimization model that can be regarded as the main engine of the Decision Support System developed by the company to achieve an optimal decision in inventory control.

## Mathematical model of the problem

In this section, we formulate a mathematical model of the decision problem. As described, we need to decide on raw material selection, delivery quantities, and compositions on the four consecutive weeks covered. In the following, we derive a mixed integer linear programming that represents the decision problem. We first present the sets, the parameters, and the decision variables used in the mathematical model. We then present the constraints which represent the production rules and capacity, followed by the discussion concerning the objective function of the optimization model. The integer linear programming is written after that.

### Sets and parameters

• M={1,2,3,4} as the set of weeks on the supply cycle,
• N as the number of raw materials,
• N={1,2,…,N} as the set of raw materials,
• I as the number of items,
• ℑ={1,2,…,I} as the number of items,
• $P\cup_{j\in M}P_{j}$ as the set of items to be produced on the planning horizon, where $P_{j}$ as the set of items to be produced on week j,
• For i∈ℑ,k∈N,
  $$f_{ik}=\left\{ \begin{array}{cc} 1, & \text{if}\;\text{item}\;i\;\text{can}\;\text{be}\;\text{produce}\;\text{by}\;\text{using}\;\text{raw}\;\text{material}\;k\\ 0, & \text{otherwise} \end{array}\right.$$
• For $i\in P_{j},g_{ijk}$ as the demand of raw material k item i on week j,
• For $j\in M,D_{j}$ as the set of the total demand on week j,
• For $k\in N,mo_{k}$ as the minimum one-year order quantity of raw material k,
• For $k\in N,c_{k}$ as the unit price of raw material k,
• For $k\in N,\sigma_{k}$ as the one-month minimum order quantity of raw material k, if purchased,
• For $k\in N,z_{0k}$ as the level of inventory of raw material k just before the first delivery on the first week,
• ss as the safety stock for each raw material at the end of each week,
• *maxcap* as the warehouse capacity,
• *hc* as the holding cost per item per week.

### Decision variables

Define:

• $\forall k\in N,x_{k}$ as the amount of raw material k purchased. $x_{k}=0$ if raw material k is not purchased, and $\sigma_{k}\leq x_{k}\leq D$ otherwise. ∀k∈N,
  $$y_{k}=\left\{ \begin{array}{cc} 0, & 0\leq x_{k}<\sigma_{k}\\ 1, & \sigma_{k}\leq x_{k}\leq D. \end{array}\right.$$

The variables $y_{k}$ are defined to handle the discontinuity property of the variables $x_{k}$.

The relationship between $x_{k}$ and $y_{k}$ are written as constraints (1) and (2).

• $\forall j\in M,\forall k\in N,{\hat{x}}_{jk}$*as the amount of raw material*k*delivered at the beginning of week*j*.*
• $\forall j\in M,\forall i\in P_{j},\forall k\in N,$
  $$a_{ijk}=\left\{ \begin{array}{cc} 1, & \text{if}\text{item}\;i\;\text{on}\;\text{the}\;\text{week}\;j\;\text{produce}\;\text{by}\;\text{using}\;\text{raw}\;\text{material}\;k\\ 0, & \text{otherwise} \end{array}\right.$$
• $\forall j\in M,\forall i\in P_{j},\forall k\in N,b_{ijk}$*as the proportion of raw material k in item i produced in week j.*
• $\forall j\in M,\forall k\in N,z_{jk}$*as the level of inventory raw material*k*at the end of week*j*.*

### Constraints

The following mathematical expressions are the constraints for our mathematical model. We write these constraints in groups where we give a concise explanation in each group are for creating them.

**Constraint I** are set to handle the discontinuity value of $x_{k}$. ∀k∈N,

(1) $$x_{k}\leq y_{k}D$$

(2) $$x_{k}\geq \sigma_{k}y_{k}$$

The relations (1) and (2) along with the definition of $y_{k}$ ensure the value $x_{k}=0$ or $\sigma_{k}\leq x_{k}\leq D$. As an illustration, suppose that we obtain a solution in which $x_{k}=\frac{1}{2}\sigma_{k}$ for some k. It follows that $y_{k}=0$, and satisfaction of (1) and (2) will yields $x_{k}=0$. This contradicts the assumption that $\sigma_{k}\leq x_{k}\leq D$.

**Constraint II** is set to fulfill the weekly allocation of each type of raw material. ∀k∈N,

(3) $$x_{k}=\sum_{j\in M}{\hat{x}}_{jk}$$

**Constraint III** is set to fulfill the raw material demand each week. ∀j∈M,

(4) $$\sum_{k=1}^{N}{\hat{x}}_{jk}+\sum_{k=1}^{N}z_{\left(j-1\right)k}\geq D_{j}$$

**Constraint IV** is set to ensure each item in $P^{2}$ is produced by using at least two raw materials. $\forall j\in M,\forall i\in P^{2}$,

(5) $$\sum_{k\in N}a_{ijk}\geq 2$$

**Constraints V** concern on the relation among $f_{ik},a_{ijk},b_{ijk},$ and $x_{jk}$. ∀j∈M,i∈P,k∈N.

(6) $$a_{ijk}\leq f_{ik}$$

(7) $$\forall j\in M,\forall i\in P,\forall k\in Nb_{ijk}\leq f_{ik}a_{ijk}$$

(8) $$\mu a_{ijk}\leq b_{ijk}$$

For a small value of μ. The explanations for the relations 6), ((7), and (8) are similar to one for relations (1) and (2).

(9) $$\forall j\in \hat{M},\forall i\in P_{j},\sum_{k\in N}b_{ijk}=1$$

Which states that the total proportion of raw materials used for item i produced on week j must be equal to 1.

**Constraints VI** are set to fulfill Policy II. $\forall j\in \hat{M},\forall i\in P_{j}^{2},k_{1},k_{2}\in N,k_{1}\neq k_{2}$

(10) $$\left(1-a_{ijk_{1}}\right)+\left(1-a_{ijk_{2}}\right)\geq b_{ijk_{1}}-b_{ijk_{2}}$$

(11) $$\left(1-a_{ijk_{1}}\right)+\left(1-a_{ijk_{2}}\right)\geq b_{ijk_{2}}-b_{ijk_{1}}$$

The relations (10) and (11) enforce that if item i produced on week j using two different raw materials $k_{1}$ and $k_{2}$ with $b_{ijk_{1}}\neq b_{ijk_{2}},$ then the left-hand sides of (10) and (11) are equal to zero meanwhile one of the right-hand side of 10 or (11) is less than zero. It concludes that $b_{ijk_{1}}$ and $b_{ijk_{2}}$ must be equal.

**Constraints VII** are set to ensure that the level of inventory just after raw material delivery does not exceed the maximum capacity. On the beginning of week 1:

(12) $$\sum_{k\in N}\left(z_{0k}+{\hat{x}}_{1k}+z_{1k}\right)-D_{1}\leq \text{maxcap}$$

(13) $$\begin{array}{ccc} & & \;\;\;\forall j\in \left\{2,3,4\right\},\\ & & \sum_{k\in N}\left(z_{\left(j-1\right)k}+{\hat{x}}_{\left(j-1\right)k}\right)-\sum_{i\in P_{j}}b_{ijk}g_{ik}+z_{jk}\leq \text{maxcap} \end{array}$$

(14) $$\begin{array}{ccc} & & \;\;\;\;\forall j\in M,\\ & & \sum_{k\in N}\left(z_{\left(j-1\right)k}+{\hat{x}}_{jk}\right)-\sum_{i\in P_{j}}b_{ijk}g_{ik}+z_{jk}\leq \text{maxcap} \end{array}$$

**Constraint VIII** is set to ensure that the level of inventory at the end of each week must be greater than or equal to the safety stock. ∀j∈M,∀k∈P,

(15) $$z_{jk}\geq ss$$

### Objective functions

**Fig. 3:**
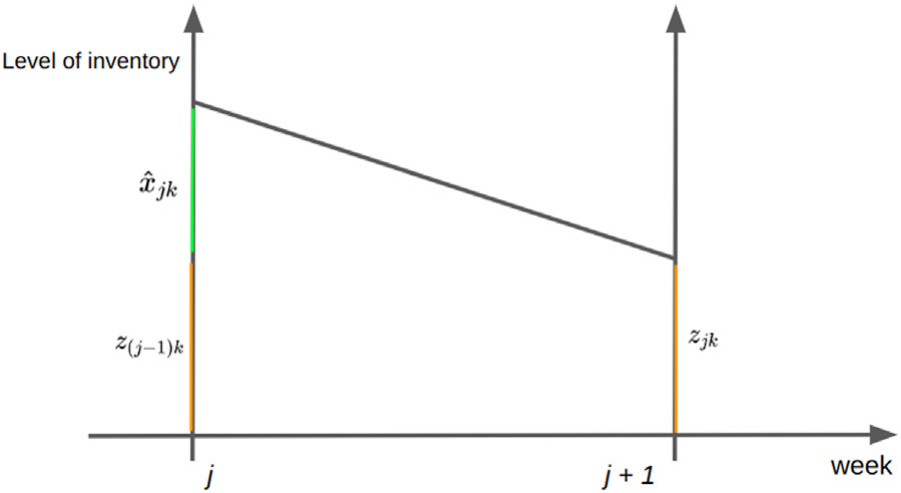
Illustration for level of inventory on the period between week j and week *j* + 1.

We define the objective function as the sum of the holding cost, the purchase cost, and a function for accommodating the one-year minimum order quantity contracts. The level of inventory of raw-material k for one week can be seen in Fig. 3.

So that the holding cost can be given as:

$$\frac{1}{2}hc\sum_{j\in M}\sum_{k\in N}\left(z_{\left(j-1\right)k}+z_{jk}+{\hat{x}}_{jk}\right)$$

Meanwhile the purchase cost can be given as:

$$\sum_{k\in N}c_{k}x_{k}$$

We consider another function in the objective function, which is created to accommodate the one-year minimum order quantity contracts.

Constraints I - VIII concern the fulfillment of the one-month minimum order quantity contract, the monthly demand, warehouse capacity constraint, safety stock constraint, and raw material composition requirements. Meanwhile, the one-year minimum order quantity contract is quite difficult to express as a constraint in the optimization model with a one-month-long planning horizon.

Therefore, we accommodate the yearly purchase contract which we represent as a part of the objective function of our optimization problem. To fulfill the one-year minimum order quantity contracts, we define a penalty function:

$$-\sum_{k\in N}\alpha_{k}mo_{k}x_{k}$$

Where $\alpha_{k}$ is multiplier constants that will be discussed later. By denoting $\bar{x}$ as the vector with elements $x_{k}$ and ${\hat{x}}_{jk}$, and $\bar{z}$ as the vector with elements $z_{jk}$ the objective function of our optimization model is then can be written as:

$$F\left(\bar{x},\bar{z}\right)=\frac{1}{2}hc\sum_{j\in M}\sum_{k\in N}\left(z_{\left(j-1\right)k}+z_{jk}+{\hat{x}}_{jk}\right)+\sum_{k\in N}c_{k}x_{k}-\sum_{k\in N}\alpha_{k}mo_{k}x_{k}$$

### Optimization model

The optimization model for supplier selection, order allocation, and raw material composition can be written as a mixed integer linear programming:

**Table:** 

**minimize**	$F(\bar{x},\bar{z})$
**Subject to**	**Constraints I - VIII**
	$x_{k},{\hat{x}}_{jk},z_{jk}\in Z^{+},y_{k},a_{ijk}\in \left\{0,1\right\},0\leq b_{ijk}\leq 1$

where the set of **Constraint I** up to **Constraints VIII** are given by equations (1) – (15).

## Method validation

In this section, we give an example of the solution of the optimization model we derived in the previous section. In this example, we consider an instant where *N* = 6 and *I* = 51, so that we have 2508 decision variables.

### Parameter values

The following Table 1 show the matrix of raw-material flexibility of all items.

**Table 1:** The flexibility matrix of items 1–51 and raw material 1–6.

Item	Raw material					
	1	2	3	4	5	6
1	1	1	0	1	0	0
2	1	1	1	1	1	1
3	1	1	1	1	1	1
4	1	1	1	1	1	1
5	1	1	0	1	0	0
6	1	1	1	1	1	1
7	1	1	1	1	1	1
8	1	1	1	1	1	1
9	1	1	0	1	0	0
10	1	1	0	1	0	0
11	1	1	1	1	1	1
12	1	1	1	1	1	1
13	1	1	1	1	1	1
14	1	1	1	1	1	1
15	1	0	0	1	0	0
16	1	0	0	1	0	0
17	1	1	1	1	1	1
18	1	1	1	1	1	1
19	1	1	1	1	1	1
20	1	1	0	1	0	0
21	1	1	0	1	0	0
22	1	1	1	1	1	1
23	1	1	1	1	1	1
24	1	1	1	1	1	1
25	1	1	1	1	1	1
26	1	1	1	1	1	1
27	1	1	1	1	1	1
28	1	1	1	1	1	1
29	1	1	1	1	1	1
30	1	1	1	1	1	1
31	1	1	1	1	1	1
32	1	1	1	1	1	1
33	1	1	1	1	1	1
34	1	1	1	1	1	1
35	1	1	0	1	0	1
36	1	1	1	1	1	1
37	1	1	1	1	1	1
38	1	1	1	1	1	1
39	1	1	1	1	1	1
40	1	1	1	1	1	1
41	1	1	0	1	0	0
42	1	1	0	1	0	0
43	1	1	1	1	0	1
44	1	1	0	1	0	0
45	1	1	0	1	0	0
46	1	1	0	1	0	1
47	1	1	1	1	1	1
48	1	1	1	1	1	1
49	0	0	0	1	0	0
50	0	0	0	1	0	0
51	1	1	0	1	0	0

Next, we present the instant of the total raw material demands during a planning horizon in Table 2.

**Table 2:** The raw material demand (in kg) of items 1–51.

*Item*	*Raw material demand in week:*			
	1	2	3	4
1	2555.1	2555.1	0	10,220.4
2	0	4300	8600	0
3	0	0	0	1800
4	0	495	0	0
5	0	0	0	0
6	3150	0	6300	0
7	0	1050	0	350
8	1650	0	0	2200
9	2080	1560	1040	0
10	0	1280	0	0
11	0	0	4200	0
12	0	2700	0	0
13	350	350	0	0
14	0	300	500	200
15	85	0	0	0
16	110	165	0	0
17	0	0	17,400	17,400
18	74,050	222,150	14,800	0
19	0	11,605	0	0
20	12,750	0	0	0
21	3500	0	14,000	14,000
22	42,100	0	10,550	0
23	0	0	5600	11,200
24	5400	5400	0	5400
25	1350	2250	0	0
26	0	32,400	43,200	0
27	10,350	31,050	0	0
28	118,200	0	0	0
29	3330	4995	0	0
30	0	1950	0	5850
31	0	6300	6300	0
32	2310	3465	0	0
33	0	2500	0	0
34	0	0	20,850	0
35	12,150	0	0	0
36	0	0	0	0
37	12,750	12,750	8500	8500
38	1550	3100	0	0
39	0	10,600	5300	21,200
40	0	0	10,450	20,900
41	9625	0	1375	1375
42	0	0	3080	0
43	0	2250	450	2250
44	0	0	14,100	0
45	6700	13,400	10,050	6700
46	6500	13,000	0	0
47	26,200	13,100	0	0
48	12,000	8000	0	12,000
49	0	126	0	0
50	1323	0	3969	0
51	1170	1170	0	780

We can see that items 5 and 36 do not have to be produced during this planning horizon. We also see that most items must be produced within only two or three weeks of this planning horizon, with varying demand. The other parameter values are given in the following Table 3.

**Table 3:** The other parameter's value.

*Parameter*	*Raw material*					
	1	2	3	4	5	6
Price	16.6	15.5	18.1	14.7	19.8	15.2
One-month min order qty (in kg)	15,000	10,000	11,500	9000	8000	12,500
Level of inventory in week 0 (kg)	3250	2850	3300	2500	3500	3050
One-year min order qty (in kg)	7956,00	19,944,00	648,000	2952,00	288,000	4212,00
Safety stock (in kg)	2500	2500	2500	2500	2500	2500

From Table 2 we know that raw material 4 must be purchased since item 49 and 50 can be produced just by using raw material 4. The price of raw material 4 is the lowest one. But the one-year minimal order quantity is the third smallest. So that we may guess that the optimal solution, $x_{4}$ will have immense value but is not the biggest one, among others. The value of one-year minimum order will be reviewed by the company every semester. However, the order proportion per supplier remains the same.

### Validation

We solve the optimization problem by creating computer codes using R language (version 4) where the optimization problem formulation and the solution technique used are referred to dplyr [1] and ompr [2] libraries.

The values of $x_{k},k\in N$ are given in the second column of Table 4 in the following. The weekly deliveries are given on columns 4 up to 6. We see that the total one-month order quantity exceeds the one-month minimum order quantity $\sigma_{k}$, so that set of Constraints I is satisfied.

**Table 4:** Total order quantity and weekly deliveries.

Raw Material	$x_{k}$	${\hat{x}}_{jk}$			
	Total order qty (in kg)	Week 1 delivery (in kg)	Week 2 delivery (in kg)	Week 3 delivery (in kg)	Week 4 delivery (in kg)
1	192,649	6553	65,295	81,722	39,079
2	543,385	354,839	23,595	96,122	68,829
3	90,742	41,092	26,550	14,400	8700
4	56,508	33,264	209	18,369	4666
5	52,958	42,608	10,350	0	0
6	202,853	70,728	111,075	0	21,050
Total	1139,094	549,083	237,074	210,613	142,324

Another decision variables, such as: ${\hat{x}}_{ijk},a_{ijk},b_{ijk},$ and $z_{jk}$ are in optimal solution. We have checked that set of Constraints II – V are satisfied with this optimal solution. For brevity, we do not present those values here. We just present and discuss some of them, to show that the remaining constraints are Satisfied.

Most values of $b_{ijk}$ are 0.5 which means most of items are produced by using a composition of two raw materials. The values of $b_{ijk}$ may vary from one week to another week, as illustrated in Table 5. The composition of raw materials for item 46 in week 1 is different from the composition in week 2. Furthermore, Table 5 shows us that the proportions of raw materials used for producing an item are the same. We have checked this property through all for all and for all so that we are sure that set of Constraints VI are satisfied.

**Table 5:** The values of $b_{ijk}$ for *i* *=* *45* and *i* *=* *46*.

*Item*	Week	$b_{ijk}$					
		1	2	3	4	5	6
45	1	0	0.5	0	0.5	0	0
45	2	0.5	0.5	0	0	0	0
45	3	0.5	0.5	0	0	0	0
45	4	0.5	0.5	0	0	0	0
46	1	0.33	0.33	0	0.33	0	0
46	2	0.5	0.5	0	0	0	0
46	3	0	0	0	0	0	0
46	4	0	0	0	0	0	0

The following Table 6 shows the composition of raw materials for items 49 and 50. We can see that these items are produced by using raw material 4, which confirms the flexibility matrix in Table 2.

**Table 6:** The values of $b_{ijk}$ for *i* *=* *49* and *i* *=* *50*.

*Item*	Week	$b_{ijk}$					
		1	2	3	4	5	6
49	1	0	0	0	0	0	0
49	2	0	0	0	1	0	0
49	3	0	0	0	0	0	0
49	4	0	0	0	0	0	0
50	1	0	0	0	1	0	0
50	2	0	0	0	0	0	0
50	3	0	0	0	1	0	0
50	4	0	0	0	0	0	0

The fulfillment of the safety stock constraint and the maximum capacity constraint (set of Constraints VII and VIII) can be seen in the following Table 7 and 8.

**Table 7:** Weekly raw material demand, delivery, and level of inventory.

Info	Week:			
	1	2	3	4
Demand	373,288	416,316	210,614	142,325
*Level of inventory* in week *j-1*	18,450	194,245	15,002	15,001
Deliveries	549,083	237,074	210,613	142,324
*Level of inventory* in week *j*	194,245	15,002	15,001	15,000

**Table 8:** the fulfillment of the safety stock constraint and the maximum capacity constraint (set of constraints VII and VIII).

Raw material	*Level of inventory* in week			
	1	2	3	4
1	2501	2501	2500	2500
2	181,743	2501	2500	2500
3	2500	2500	2500	2500
4	2500	2501	2501	2500
5	2500	2500	2500	2500
6	2500	2500	2500	2500

Note that the objective function's definition, which accommodates the one-year minimum order quantity contract, yields a balancing of purchase price criteria and the one-year minimum order quantity criteria. In the following table, we represent the optimal solutions obtained using different objective functions. Notice that the total raw materials purchased in all solutions are different.

• Objective function I:
  $$\frac{1}{2}hc\sum_{j\in M}\sum_{k\in N}\left(z_{\left(j-1\right)k}+z_{jk}+{\hat{x}}_{jk}\right)$$
• Objective function II:
  $$\frac{1}{2}hc\sum_{j\in M}\sum_{k\in N}\left(z_{\left(j-1\right)k}+z_{jk}+{\hat{x}}_{jk}\right)+\sum_{k\in N}c_{k}x_{k}$$
• Objective function III:
  $$\frac{1}{2}hc\sum_{j\in M}\sum_{k\in N}\left(z_{\left(j-1\right)k}+z_{jk}+{\hat{x}}_{jk}\right)+\sum_{k\in N}c_{k}x_{k}-\sum_{k\in N}\alpha_{k}mo_{k}x_{k}$$

From Table 9 we see if we just use the purchase price in the objective function, raw material 4 is purchased with the biggest amount. But since the distribution of $mo_{k}$ raw material 4 has the second smallest value, then if we consider this distribution in the objective function, raw material 4 is purchased with a smaller amount. Total purchased price from all solutions are acceptable for business. But solution obtained by objective function III has the lowest total purchase price.

**Table 9:** Comparison among order quantities obtained by using different objective functions.

Raw material	Optimal solution $x_{k}$ obtained by objective function:		
	I	II	III
1	221,764	416,549	192,649
2	129,283	474,751	543,385
3	226,236	83,116	90,742
4	142,492	35,051	56,508
5	198,872	88,378	52,958
6	300,491	41,249	202,853
Total	1219,138	1139,094	1139,094

**Fig. 4:**
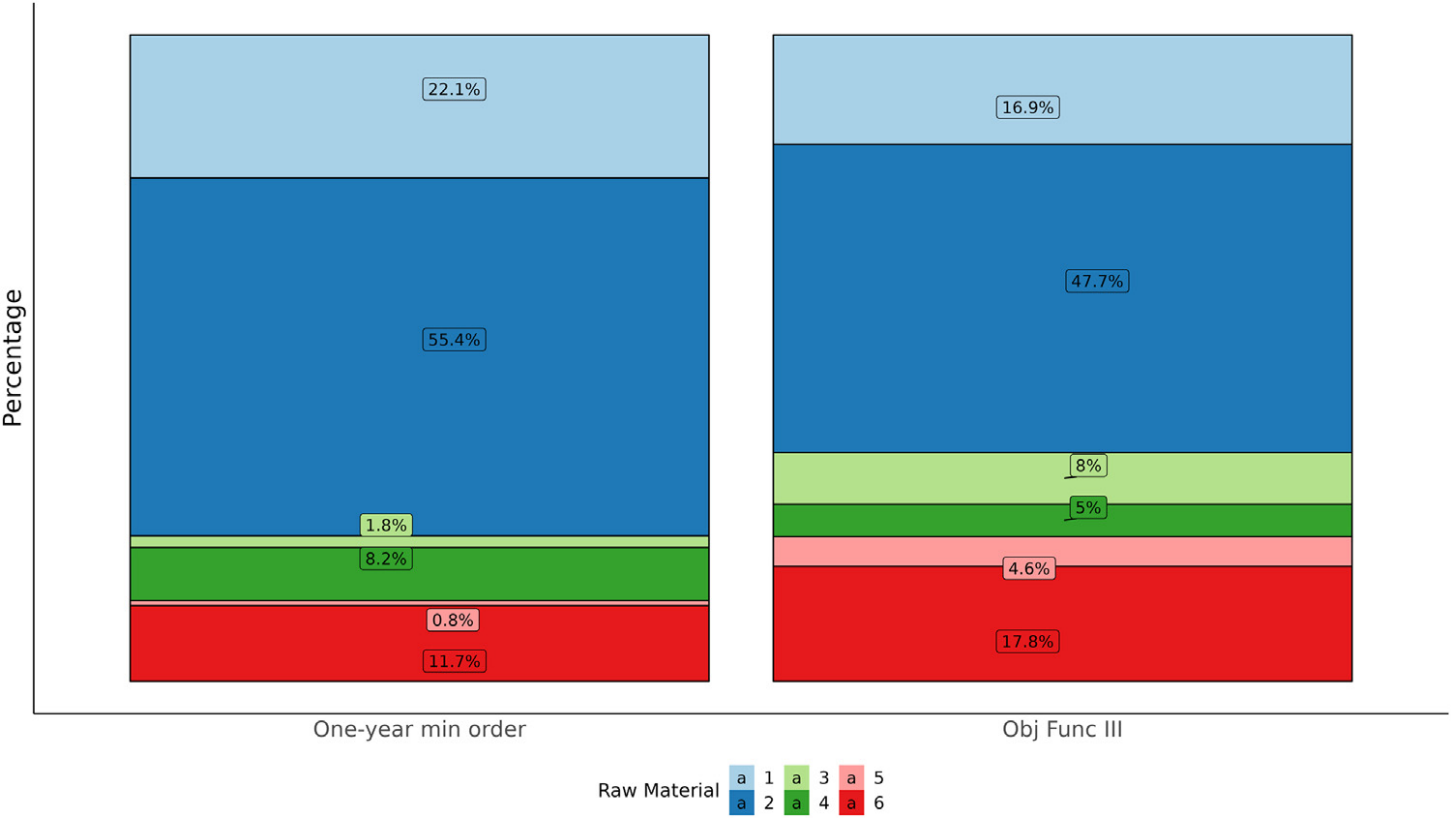
Comparison between the one-year minimum order quantities and the optimal order quantities for the instant problem by using objective function III.

In the following Fig. 4, we compare the distribution of one-year minimum order quantities with the distribution of one-month order quantities obtained by using objective function III. This comparison shows that objective function III is effective for finding order quantities whose distribution is similar to distribution of one-year minimum order quantities.

The last thing to be discussed is the multiplier parameter $\alpha_{k}$. These parameters should be set as a positive number in the early months of the year, when the one-year minimum order quantity contracts are still far away from being fulfilled. As soon as the contract for raw material k is fulfilled, we can set $\alpha_{k}=0$ for some k.

We performed several numerical experiments to check the optimal solutions for various demands and objective functions. From this experimentation, we are sure that our MILP gives the desired optimal solutions.

## Conclusion

In this paper, we present a mixed-integer linear program (MILP) for raw-material selection and composition, and an order allocation problem faced by a beverage company. We performed several numerical experiments to check the optimal solutions for various demands and objective functions. Throughout all our experimentations, the optimization model we derived consistently provides solutions that satisfy all constraints. This gives us great confidence in having derived an optimization problem that accurately represents the challenges faced by the company. It also should be noticed that dplyr [1]package R programming language can handle data manipulation faster and in an easier way. So, if some constraints are changed or there are some new constraints that have to be added, the use of dplyr package will not give us any difficulty in handling data manipulation as long as we have performed an appropriate modification. Meanwhile, the ompr [2] package can model MILP in an algebraic way directly in R and offers the possibility to solve a model with different solvers. Both packages produce optimal solutions efficiently, only requiring on average 50.8 s for searching the problem, which involves 2508 decision variables.

We realize that the search time for the optimal solution will increase as the number of decision variables increases. Due to the lack of similar large-sized problems faced by the other companies, we have not conducted numerical experimentation on larger problem sizes so that we cannot say anything about the performance of dplyr and ompr packages in solving large-sized problems.

It should be noticed that the definition of objective function III which accommodates the one-year minimum order quantity contract yields a balance of purchase price criteria and the one-year minimum order quantity criteria. In the following table we represent the optimal solutions obtained by using different objective functions.

## Supplementary material *and/or* additional information

**Introduction and Supporting Articles**

This paper concerns the derivation of the optimization model, which can be categorized as a multi-product multi-period multi-supplier raw material selection and composition, and order quantity problem. The multi-product multi-period multi-supplier raw material selection problem has been addressed in several articles such as Sambatt, Woarawichai, and Naenna [3], but they do not address the minimum one-year order quantity contracts so that their optimization problem is simpler than our optimization problem. In general, our optimization problem is much more complex compared to the one criterion supplier selection studied in an enormous articles such as Reck & Long [4], Monckza & Trecha [5], & Porter, and Harding papers. Later, supplier selection research has developed into a problem with multiple criteria, such as criteria for quality of goods, on-time delivery, and after-sales service, as well as environmental and socio-political criteria for suppliers (see Smytka & Clemens [6], Gray [7]). What is interesting is that, in general, these criteria contradict each other. For example, goods offered at low prices (positive values for the price criteria) may have negative values for on-time delivery criteria. The complexity of this issue is compounded by the fact that some criteria are quantitative (price, timeliness of delivery, specification/quality of goods, etc.), but other criteria are qualitative (after-sales service, environmental and socio-political criteria of suppliers).

The paper by Weber Current & Benton (Co Ao [8]) is a paper at the beginning of this research on multi-criteria supplier selection, which presents research results with four criteria, namely Price, Quality, Delivery and Service (PDQS). This paper together with Hurkens, van der Valk, Wynstra [9] introduces the supplier selection problem under the concept of Total Cost Ownership (TCO), a financial analysis tool to examine the direct and indirect costs of a product's production. These direct and indirect costs then become the criteria in the supplier selection process. These papers on TCO include Ferrin & Plank [10], Degraeve & Roodhooft [11]. Our optimization problem is categorized as a multi-criterion one, where one of the criteria is a new one, i.e. the minimum one-year order quantity.

After the rise of conceptual research on supplier selection with multi-criteria, then we quite easily find a proposal to use the Analytic Hierarchy Process (AHP), a decision-making method when it comes to ranking of many criteria (see Dyer [12]), as a method of solving supplier problems. selection. AHP provides a framework for addressing various criteria involving intuitive, rational, qualitative, and quantitative aspects. Other papers that discuss the AHP approach to supplier selection solutions include Bard, Belton [13], Bhutta & Huq [14], Nydick & Hill [15].

Another method proposed as a solution to the supplier selection problem is an optimization method or mathematical programming as proposed by Degraeve & Roodhooft [11], Khalifa & Mohammed Al-Shabi [16], and Nispeling [17]. A special optimization method, namely multi-objective goal programming, was proposed by Weber & Ellram (C. A. [18]). Multi-objective programming is very suitable to be used to resolve conflicts between existing criteria and the existence of just-in-time scenarios. Meanwhile, Masella & Rangone [19] offer a dynamic programming method as a method of completing this supplier selection, where input variables are set as controls and environmental variables and status variables are set as the internal workings of the organization, and output variables are seen as company performance. Another optimization method used as a solution method is Data Envelopment Analysis (DEA), as proposed in the paper of Pitchipoo, et al. [20] and Shahrzad, et al. [21].

Apart from these methods, we get the combined use of the two methods above (hybrid method), such as the one proposed by Li, Wong, & Kwong [22] which combines the AHP method and multi-objective programming. Another approach is the metaheuristic method proposed by Alejo-Reyes, et al. [23].

## Ethics statements

None.

## CRediT authorship contribution statement

**Mohammad Rizka Fadhli:** Conceptualization, Resources, Data curation, Software. **Saladin Uttunggadewa:** Conceptualization, Methodology, Writing – review & editing. **Rieske Hadianti:** Methodology, Writing – original draft, Validation, Investigation. **Sri Redjeki Pudjaprasetya:** Supervision.

## Declaration of Competing Interest

The authors declare that they have no known competing financial interests or personal relationships that could have appeared to influence the work reported in this paper.

## Data Availability

Data will be made available on request.
